#  Chief Resident Election of Emergency Department (CREED) – An innovative approach to fair and bias-free chief resident selection in a residency program

**DOI:** 10.12669/pjms.38.6.6099

**Published:** 2022

**Authors:** Shahan Waheed, Noman Ali

**Affiliations:** 1 Dr. Shahan Waheed, MBBS, MD, FCPS (EM), Assistant Professor Department of Emergency Medicine, Aga Khan University Hospital, Stadium Road, Karachi, Pakistan; 2 Dr. Noman Ali, MBBS, FCPS (EM), Assistant Professor, Department of Emergency Medicine, Aga Khan University Hospital, Stadium Road, Karachi, Pakistan

**Keywords:** Residency, Emergency Medicine, Pakistan

## Abstract

Emergency medicine has transitioned from developing to a developed specialty in Pakistan. It is gaining recognition in the national and international arena. Residency in emergency medicine is a tough pathway in which a resident learns and is assessed at multiple levels. The attributes that are needed for an empathetic emergency medicine physician are multifaceted. Chief resident selection has been an important step in postgraduate residency. The selection process was traditionally based on seniority and academic achievements with no consideration for soft skills. In the current write-up, we are proposing an evidence-base sequential chief resident selection process called Chief Resident Election of Emergency Department (CREED). The program was developed keeping in consideration the traditional method of election and interviews with the incorporation of reflexive, leadership, communication, and collaboration skills.

Emergency medicine in Pakistan has moved from developing to a developed specialty that is gaining momentum on the national and international canvas.^1^ The selection of chief resident in a residency program is a stepping stone to a prosperous career that has a long-established role.^2^ It is a position that is achieved based on seniority and demands attributes of leadership, collaboration, communication, and critical thinking. The importance of this role in emergency medicine is more important in a developing country residency program where the candidate must oversee multiple fronts like; the physical & mental well-being of peers, academics, career prospects, and weak health systems infrastructure that gives the reason for making selection process looking not just academics or seniority-based but other non-clinical skills as well.^3^ Additionally, the process needs to be fair with minimal bias in the assessment of soft skills. The selection process has been given importance in the developed country residency program with some evidence shown in the literature.^4^ In this commentary, we are proposing a novel chief resident selection process that incorporates validated tools which help in assessing critical thinking, collaboration, and leadership styles, combined with candidate nominations through the use of an online survey instrument. The selection process is pilot tested and is sought to provide a bias-free and fair chance of selection and evaluation of necessary skills that are needed for this leadership position during postgraduate training.

The Chief Resident Election of Emergency Department (CREED) consists of four sequential steps (Fig.1). In the first step, there was an online survey made on the direct poll. This browser was chosen to avoid duplication of responses. Furthermore, a unique identifier was allotted to each section of the survey so that only a single response can be given. The direct poll encompasses responses from residents, faculty, nurses, and administrative staff. A pre-defined weightage was given to each of the four domains of the survey. In the second step, the top three candidates were selected based on the survey results for an interview with a reflexive writing piece on “Why I should be a chief resident”. The reflexive writing piece was meant to review the vision of the selected candidate during his/her tenure as a chief resident. The reflexive piece was evaluated by an independent person not involved in the selection process. This activity was also meant to assess the writing skills of a chief resident. In the third step before the interview, candidates were assessed in terms of pre-validated questionnaires on leadership, collaboration, and communication skills. This step had no part in the grading of the selected individuals but was meant to assess the qualities that are pivotal in leadership roles. This assessment aimed to work on developing these important attributes of leadership. The fourth and final step was face to face interview that was undertaken by the program director and associate program director to judge the candidates based on a checklist that assessed their soft skills, vision, and purpose for this important administrative role.

**Fig.1:**
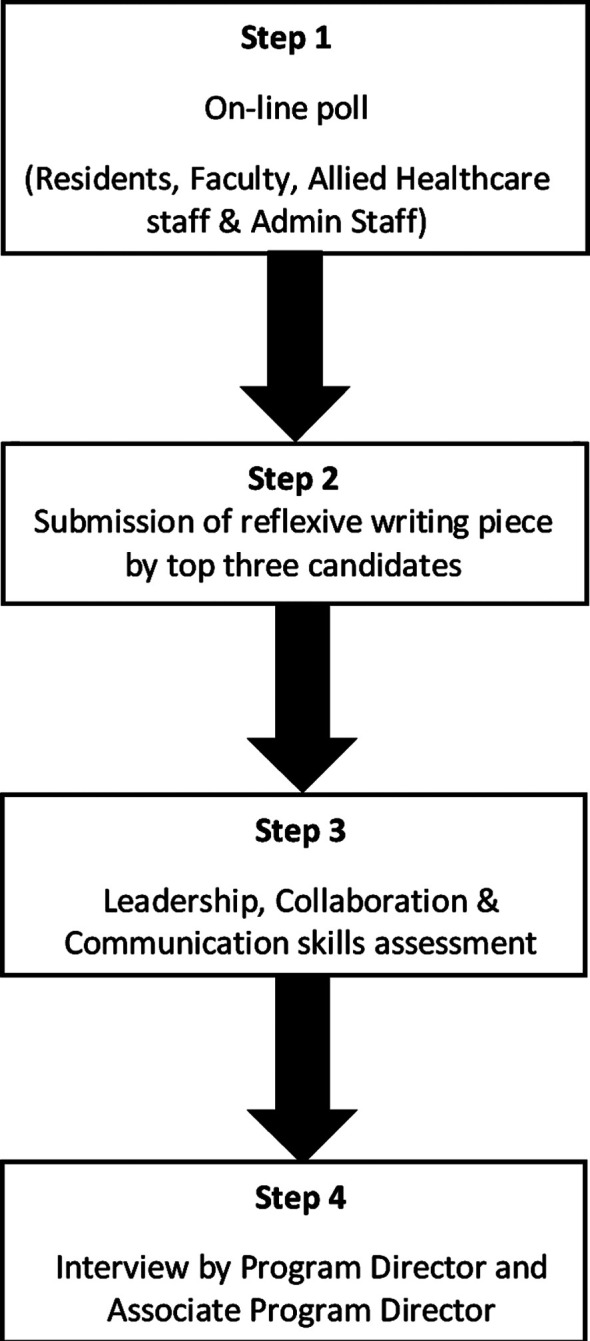
Four-step selection process for a chief resident in an emergency medicine residency program.

Our chief residency selection process was developed based on the evidence of leadership skills needed by senior-level emergency medicine faculty. These attributes assessment in a systematic way is necessary, as it helps the clinical supervisor and residency program to work with a direction. Chief residents are the future leaders and being a relatively new specialty that is making its mark on the national and international canvas, addressing this important aspect at the very beginning of their professional careers may help the upcoming faculty to work with a vision. Each distinctive process may help the leadership in assessing candidates independently. Additionally, the evaluation of the attributes by an independent person who was not involved in the selection process may help to reduce the bias that prevails in the selection process. This is the first time that any residency program in the country is showcasing its chief resident selection process so that other developing programs can adapt and innovate on their existing selection methodology.

## Authors’ Contributions:

SW: Conceived the idea and developed the initial draft of the paper.

NA: Prepared the final draft and is accountable for the accuracy and integrity of work.

All authors have read and approved the final manuscript.
